# Two-dimensional speckle-tracking echocardiography for evaluation of dilative ascending aorta biomechanics

**DOI:** 10.1186/s12872-016-0434-9

**Published:** 2017-01-13

**Authors:** Monika Bieseviciene, Jolanta Justina Vaskelyte, Vaida Mizariene, Rasa Karaliute, Vaiva Lesauskaite, Raimonda Verseckaite

**Affiliations:** 1 Department of Cardiology, Medical Academy, Lithuanian University of Health Sciences, Eivenių Str. 2, Kaunas, 50009 Lithuania; 2 Institute of Cardiology at Lithuanian University of Health Sciences, Kaunas, Lithuania

**Keywords:** Ascending aorta, Biomechanics of aorta, Speckle-tracking echocardiography, Stiffness

## Abstract

**Background:**

Two-dimensional (2D) echocardiography is one of the most feasible, noninvasive methods for assessing the aortic diameter and biomechanical changes. We studied possible interfaces between noninvasive biomechanical and speckle-tracking (ST) echocardiographic data from dilated aortas.

**Methods:**

Altogether, 44 patients with dilative pathology of ascending aorta (DPAA) were compared with subjects without ascending aortic dilation (diameter <40 mm). DPAA patients formed two groups based on diameter size: group 1, ≤45 mm diameter; group 2, >45 mm. Conventional and 2D-ST echocardiography were performed to evaluate peak longitudinal strain (LS), longitudinal (LD) and transverse (TD) displacement, and longitudinal velocity (VL). Aortic strain, distensibility, elastic modulus, stiffness index β of Valsalva sinuses and ascending aorta were also evaluated. SPSS version 20 was used for all analyses.

**Results:**

All linear diameters of the ascending aorta were increased in group 2 (>45 mm diameter) (*p* < 0.05). LD of the anterior aortic wall (*p* < 0.05) and TD of both aortic walls (*p* < 0.001) were least in group 2. VL of the posterior and anterior walls diminished in group 2 (*p* = 0.01). Aortic strain and distensibility were least (*p* = 0.028 and *p* = 0.001, respectively) and elastic modulus and stiffness index β values were greatest in group 2, although without statistical significance.

**Conclusions:**

Ascending aortas of both DPAA groups had reduced elasticity and increased stiffness. The greatest changes in biomechanical parameters occurred in ascending aortas >45 mm. Longitudinal ascending aortic wall motion was mostly impaired in patients with aortas >45 mm (i.e., anterior aortic wall LD, VL of the posterior and anterior walls. TD of the posterior and anterior aortic walls was significantly lower in >45 mm aortic diameter patients. TD of 5.2 mm could predict aortic dilation >45 mm (area under the curve 0.76, *p* < 0.001, confidence interval 0.65–0.87; sensitivity 87%; specificity 63%). Greater aortic dilation is associated with reduced aortic stiffness parameters and increased elastic modulus and stiffness index β. Lower LD and LS were associated with less aortic strain and distensibility. There were no significant differences in 2D-ST echocardiographic or stiffness parameters between patients with tricuspid or bicuspid aortic valves.

## Background

Aortic dilation is the most frequent pathology of the ascending aorta and a well-known risk factor for dissection. An aneurysm has been defined as localized dilation of an artery, with at least a 50% increase in the diameter compared with the expected normal diameter. Because of the increased risk of aortic dissection, surgical repair is recommended for patients with an ascending aorta aneurysm [1].

According to the 2014 European Society of Cardiology guidelines on the diagnosis and treatment of aortic disease, surgery is indicated for an aortic root aneurysm in the following patients: those with Marfan syndrome (IC class) with a maximum aortic diameter of 50 mm; those with Marfan syndrome with a 45 mm aortic diameter and risk factors; those with a 50 mm aortic diameter, a bicuspid valve, and risk factors; those with a 55 mm aortic diameter and no elastopathy [2].

The possibility of defining biomechanical properties of an aortic wall more precisely has increased in recent years, which could help improve our understanding of aortic wall properties and the potential risk for rupture [3]. Two-dimensional (2D) echocardiography is one of the most feasible and the oldest method for diagnosing this pathology. More precisely, it can be used not only for diameter assessment but also for noninvasive measurement of biomechanical changes in the aorta.

Arterial stiffness parameters (e.g., distensibility, stiffness β, an elastic modulus, aortic strain) can also be evaluated by 2D echocardiography. Arterial stiffness is one of the earliest detectable manifestations of adverse structural and functional changes in the vessel wall [4]. Arterial stiffness, which increases with age [5], is an important predictor of cardiovascular morbidity and mortality [6]. Changes in arterial stiffness may have global consequences (i.e., affecting the whole arterial system, such as age-related stiffening). Changes could also be more localized, however, as in the case of arterial pathology (e.g., an aortic aneurysm) [7].

A novel method—2D speckle-tracking echocardiography (2D-ST)—is a bedside approach to assessing human aortic wall strain and motion. Up to now there have been few data available regarding the use of 2D-ST echocardiography for evaluating the ascending aorta.

2D-ST echocardiography is able to identify specific acoustic markers (i.e., speckles) in grey-scale images and track them frame-by-frame throughout the cardiac cycle. The results enable independent calculations of motion and deformation variables, such as velocity, displacement, strain (e), and strain rate [8, 9].

We therefore investigated the elastic and mechanical properties of the ascending aorta using the novel 2D-ST method. The aim was to find interfaces between noninvasively measured biomechanical data from a dilated aorta using different echocardiographic techniques.

## Methods

### Study population

The current study included 44 patients with dilative pathology of the ascending aorta (DPAA; 78.3% men; mean age 55.41 ± 14.69 years) and 53 controls (51.9% men; mean age 58.64 ± 7.50 years). The control group consisted of subjects without ascending aorta dilation (diameter >40 mm). The DPAA group was subdivided into two groups according to the diameter of the ascending aorta: group 1, ≤45 mm diameter; group 2, >45 mm.

All DPAA patients were treated in the Cardiology Department at the Hospital of Lithuanian University of Health Sciences during 2012–2014. Inclusion criteria were an ascending aorta or sinus dilation ≥40 mm with or without aortic valve pathology, good-quality echocardiographic images, sinus rhythm, and the absence of hemodynamically significant coronary artery disease shown by coronary angiography (stenosis <50%). We excluded patients with acute aortic syndrome, aortic coarctation, an implanted pacemaker, atrial fibrillation/flutter, and/or a history of coronary artery bypass surgery.

Clinical evaluation and 2D and 2D-ST echocardiography were performed prior to cardiac surgery. All participants gave their informed written consent, and the Kaunas Regional Biomedical Research Ethics Committee approved the study (ref. number BE-2-12).

### 2D and 2D-ST echocardiography

Both conventional (2D) and 2D-ST echocardiography were performed using a Vivid 7 (GE-Vingmed Ultrasound AS, Horten, Norway) ultrasonography machine with a M3S probe. M-mode, 2D, Doppler, and 2D-ST echocardiography examinations were performed to obtain standard parasternal and apical views. To minimize the variability of measurements, all echo/Doppler evaluations were performed and analyzed by a single physician. The linear data were measured and averaged over three heartbeats. The diameters of the heart chambers and aortic diameters were measured according to the last published guidelines for cardiac chamber quantification in adults. The left ventricular (LV) volumes and ejection fractions were calculated from apical two- and four-chamber views using the biplane Simpson’s disc summation method. Aortic diameters were measured as absolute values and as values indexed to the body surface area. The aortic diameters ratio was determined as ratio of the observed diameter to expected diameter [10].

For the aortic 2D-ST analysis, tissue harmonic images were used. The mean frame rate was adjusted to 50–90 frames per second, and the cine-loop of three consecutive heartbeats was stored and transferred to a workstation for off-line ST analysis (EchoPac PC; GE Vingmed). Before analysis, the timing of an aortic valve and a mitral valve opening and closing was assessed using pulsed-wave Doppler recordings of aortic and transmitral flow, respectively.

The echocardiographic images of the ascending aorta were obtained from the long-axis parasternal view. The shortest distance from the aortic annulus with good visibility was 3 cm. Manual tracing of the inner contour of the aortic root and the proximal ascending aorta was performed during systole. Dedicated software tracked the contour on all other frames automatically. Because the tracking software was created for use in the left ventricle, we used the two-chamber analysis option and then changed the six segments to four, thus dividing the aortic wall into four segments: anterior sinus, anterior ascending aorta, posterior sinus, posterior ascending aorta. For the aortic evaluation, we excluded the “LV apical segment” analysis. After manual correction of the region of interest, correct tracking was verified.

**Fig. 1:**
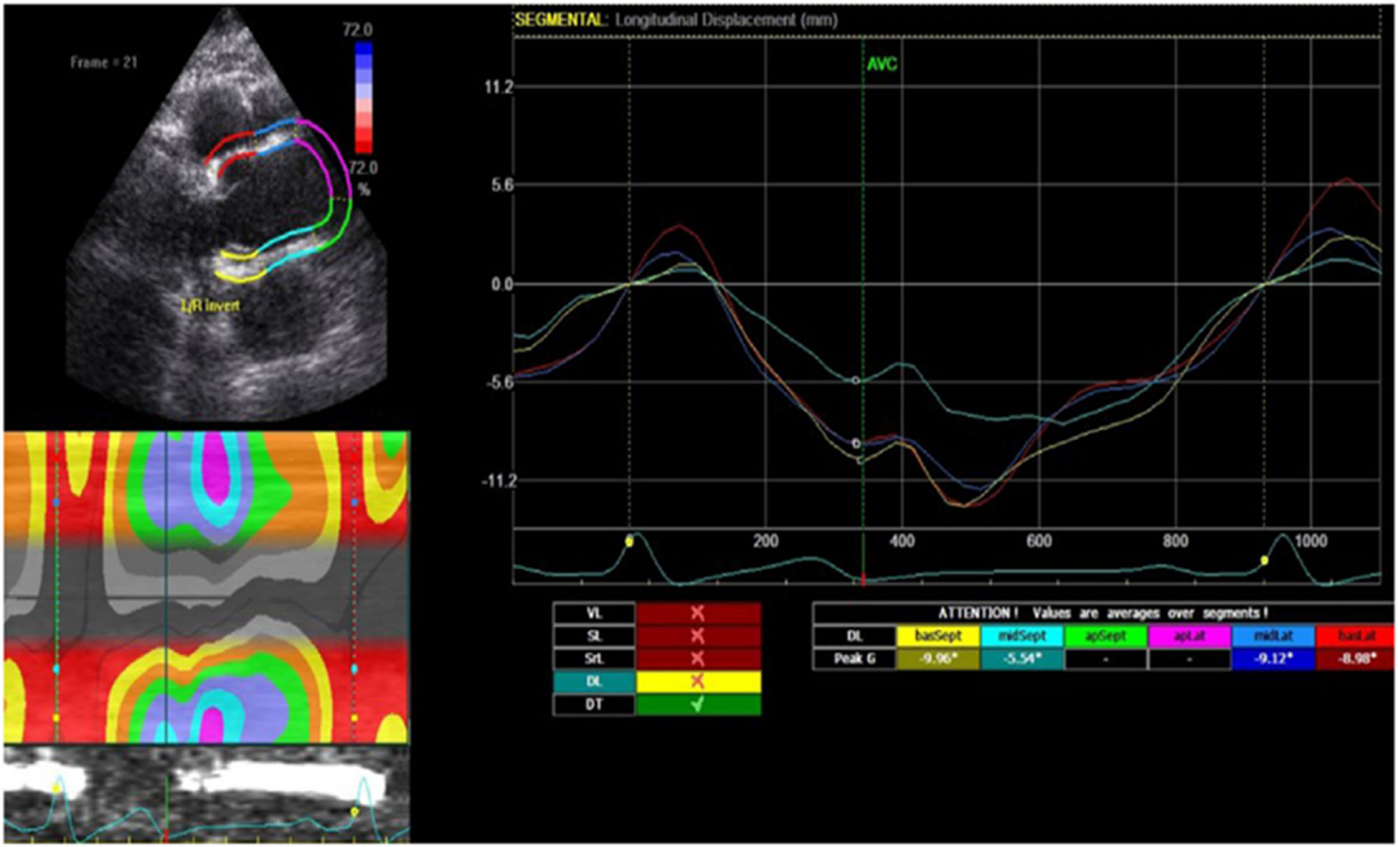
Example parasternal long axis view of a two-dimensional speckle-tracking echocardiographic image of longitudinal displacement of the ascending aorta

We then evaluated the peak longitudinal strain (LS), which is considered the change in segment length/resting segment length—i.e., deformation of an object relative to its original length (a dimensionless ratio expressed as a percent); longitudinal (LD) and transverse (TD) displacements, defined as a change in the position of an object; and longitudinal velocity (VL), displacement of an object per time unit—of the Valsalva sinuses and the ascending part of the aorta (Fig. 1).

For all of the subjects, arterial blood pressure was measured using a sphygmomanometer at the brachial artery. Aortic stiffness describes the elastic resistance that the aorta sets against distension [7]. The parameters of the ascending aorta stiffness and elasticity were calculated using previously described formulas [11, 12].

- Aortic diameter change (mm): SD − DD
- Aortic strain: (SD − DD)/DD
- Aortic distensibility: 2 × (change in aortic diameter)/(diastolic aortic diameter) × (change in aortic pressure)
- Elastic modulus: (SBP − DBP)/strain
- Stiffness index β: ln (SBP/DBP)/strain

where DD is the diastolic aortic diameter, SD is the systolic aortic diameter, DBP is the diastolic blood pressure, SBP is the systolic blood pressure, and ln is a natural logarithm.

### Statistical analysis

SPSS version 20 (SPSS, Chicago, IL, USA) was used for all data analyses. The continuous variables are expressed as the mean ± standard deviation (SD). Normal distribution of the continuous values was assessed by the Kolmogorov − Smirnov test. The categorical variables are expressed as absolute numbers and percentages. The statistical test used in these cases was the χ^2^ test. The means were compared using the two-tailed unpaired Student test or Mann–Whitney test. The relation between the variables was estimated using Pearson’s correlation (r). A receiver operating characteristic (ROC) curve was used to determine the sensitivity and specificity of strains for predicting a dilated aorta. A value of *p* < 0.05 was regarded as indicating statistical significance.

## Results

The demographic, clinical, and conventional echocardiographic data of the study groups are shown in Tables 1 and 2. More men were enrolled in the study, but there were no differences between the sex distribution, age, or body surface area in the groups. More hypertensive patients (89.3%) were included in group 2 than in group 1 or the controls. Systolic blood pressure was highest in group 2, and diastolic blood pressure was highest in the controls.

**Table 1:** Demographic and clinical data for the study population

Parameter	Controls	Diameter of ascending aorta		*p*		
		Group 1 (≤45 mm)	Group 2 (>45 mm)	Controls vs. group 1	Controls vs. group 2	Group 1 vs. group 2
Age (years)	58.2 ± 7.1	52.9 ± 13.8	60.1 ± 12.2	NS	NS	NS
Sex (F/M) (%)	43.3/56.7	18.8/81.2	25.0/75.0	NS	NS	NS
Body surface area (m^2^)	1.91 ± 0.2	2.01 ± 0.2	2.0 ± 0.2	NS	NS	NS
Arterial hypertension (%)	65.2	60.0	89.3	NS	0.02	0.02
Blood pressure (mmHg)						
Systolic	134.6 ± 13.7	138.8 ± 17.7	145.8 ± 23.5	NS	0.01	NS
Diastolic	84.1 ± 9.6	73.4 ± 13.0	78.8 ± 11.8	0.003	0.03	NS
Antihypertensive Rx (%)						
β-Blockers	25.5	85.7	77.8	<0.05	0.001	NS
ACE-I	22.0	64.3	55.6	<0.05	0.002	NS
ARB	17.6	7.7	18.5	NS	NS	NS
Smokers (%)	28.8	0	7.4	<0.05	0.02	NS
Aortic valve pathology (%)						
Aortic valve insufficiency	0	50.0	53.8	<0.001	<0.001	NS
Stenosis	0	6.2	15.4	NS	0.002	NS
Bicuspid	0	62.5	30.4	0.001	0.001	0.04

*ACE-I* angiotensin-converting enzyme inhibitors, *ARB* angiotensin receptor blockers, *NS* not significant, *Rx* treatment, Values are mean (SD) unless otherwise indicated

**Table 2:** Conventional echocardiographic data for the study population

Parameter	Controls	Diameter of ascending aorta		*p*		
		Group 1 (≤45 mm)	Group 2 (>45 mm)	Controls vs. group 1	Controls vs. group 2	Group 1 vs. group 2
LV diastolic diameter (mm)	46.9 ± 4.6	55.9 ± 7.3	55.3 ± 8.9	<0.001	<0.001	NS
LVEDDi (mm/m^2^)	24.1 ± 2.2	28.0 ± 3.9	27.5 ± 4.2	<0.001	<0.001	NS
Interventricular septum (mm)	10.7 ± 1.5	12.5 ± 1.6	13.5 ± 2.3	<0.001	<0.001	NS
LV posterior wall (mm)	10.1 ± 1.4	11.1 ± 1.5	11.9 ± 2.0	0.02	<0.001	NS
LV ejection fraction (%)	57.2 ± 2.1	54.6 ± 3.4	50.2 ± 8.9	NS	<0.05	0.029
Ascending aorta diameter (mm)						
Aortic annulus	22.5 ± 3.2	27.8 ± 3.2	26.5 ± 2.8	<0.001	<0.001	NS
Sinus at Valsalva level	35.0 ± 3.6	44.8 ± 7.6	47.2 ± 8.2	<0.001	<0.001	NS
Sinotubular junction	30.2 ± 3.1	37.6 ± 5.3	43.1 ± 8.8	<0.001	<0.001	NS
Ascending aorta in end-diastole	33.9 ± 3.3	42.8 ± 2.8	53.2 ± 6.0	<0.001	<0.001	<0.001
Cross sectional area in systole	8.6 ± 1.6	14.5 ± 1.7	21.7 ± 6.7	<0.001	<0.001	<0.001
Cross sectional area in diastole	7.7 ± 1.5	12.8 ± 1.6	19.9 ± 6.3	<0.001	<0.001	<0.001
Aortic diameter/BSA (mm/m^2^)						
Aortic annulus	11.6 ± 1.7	13.8 ± 1.5	13.4 ± 1.4	<0.001	<0.001	NS
Sinus at Valsalva	18.2 ± 1.9	22.2 ± 2.9	23.5 ± 3.6	<0.001	<0.001	NS
Sinotubular junction	15.6 ± 1.5	18.6 ± 1.5	21.7 ± 4.0	<0.001	<0.001	0.01
Ascending aorta in end-diastole	17.3 ± 1.7	21.5 ± 2.8	27.0 ± 5.5	<0.001	<0.001	<0.001
Aortic diameter index (ratio): observed/expected values						
Aortic annulus	0.89 ± 0.1	1.0 ± 0.1	1.02 ± 0.1	<0.001	<0.001	NS
Sinus at Valsalva level	1.0 ± 0.1	1.29 ± 0.2	1.35 ± 0.2	<0.001	<0.001	NS
Sinotubular junction	1.0 ± 0.1	1.24 ± 0.1	1.44 ± 0.2	<0.001	<0.001	0.01
Ascending aorta in end-diastole	1.1 ± 0.1	1.4 ± 0.2	1.76 ± 0.3	<0.001	<0.001	<0.001

*BSA* body surface area, *LV* left ventricular, *LVEDDi* left ventricular end-diastolic diameter index, Values are mean (SD) unless otherwise indicated

The LV diameters did not differ between the DPAA groups but were significantly smaller in the controls. Statistically significantly larger LV ejection fractions were observed in group 1 and the controls. The linear absolute and indexed values of diameters of the ascending aorta were largest in group 2.

There were no aortic valves with hemodynamically significant stenosis or insufficiency in the control group. In contrast, severe aortic stenosis was diagnosed in 6.2% of patients in group 1 and in 15.4% in group 2. Overall, 50% of group 1 and 53.8% of group 2 patients had severe aortic insufficiency. There was no difference in the frequency of aortic regurgitation between the two DPAA groups. All of the control patients had an anatomically tricuspid aortic valve, whereas 62.5% of group 1 patients and 30.4% of group 2 patients had a bicuspid aortic valve (BAV) (*p* < 0.04). The2D-ST echocardiographic parameters of the ascending aorta of the study groups are shown in Table 2. 2D-ST echocardiographic parameters of the ascending aorta of the study groups are shown in Table 2. The 2D-ST echocardiographic parameters of the ascending aorta of the study groups are shown in Table 3.

**Table 3:** Speckle-tracking echocardiographic parameters of the ascending aorta

Parameter		Diameter of ascending aorta		*p*		
	Controls	Group 1 (≤45 mm)	Group 2 (>45 mm)	Controls vs. group 1	Controls vs. group 2	Group 1 vs. group 2
Longitudinal displacement (mm)						
Aortic posterior sinus	−5.3 ± 4.0	−6.2 ± 4.4	−4.3 ± 3.7	NS	NS	NS
Posterior ascending aorta	−3.3 ± 3.1	−2.6 ± 4.4	−3.3 ± 3.0	NS	NS	NS
Anterior ascending aorta	−9.8 ± 4.3	−11.5 ± 5.3	−6.8 ± 5.3	NS	0.01	0.018
Aortic anterior sinus	−11.7 ± 6.1	−13.9 ± 6.5	−9.6 ± 5.2	NS	NS	0.04
Aortic PW	−4.3 ± 3.4	−4.4 ± 4.1	−3.7 ± 2.8	NS	NS	NS
Aortic AW	−10.7 ± 4.9	−12.7 ± 5.7	−8.2 ± 4.8	NS	0.042	0.02
Transverse displacement (mm)						
Aortic posterior sinus	5.7 ± 2.0	3.8 ± 3.0	3.6 ± 2.0	0.009	<0.001	NS
Posterior ascending aorta	5.4 ± 1.9	6.5 ± 3.0	4.0 ± 1.8	NS	0.002	0.003
Anterior ascending aorta	−5.6 ± 1.9	−5.7 ± 2.0	−3.9 ± 3.1	NS	0.01	NS
Aortic anterior sinus	−8.8 ± 2.2	−9.5 ± 2.6	−6.3 ± 4.1	NS	0.01	0.02
Aortic PW	5.6 ± 1.7	6.2 ± 2.1	3.8 ± 1.8	NS	<0.001	0.002
Aortic AW	−7.2 ± 2.0	−6.8 ± 2.5	−5.4 ± 2.9	NS	0.003	NS
Longitudinal strain (%)						
Aortic posterior sinus	18.9 ± 19.1	19.8 ± 10.3	13.8 ± 16.4	NS	NS	NS
Posterior ascending aorta	17.8 ± 13.8	17.6 ± 14.6	13.54 ± 9.9	NS	NS	NS
Anterior ascending aorta	23.0 ± 17.6	29.0 ± 17.1	19.5 ± 10.9	NS	NS	NS
Aortic anterior sinus	12.5 ± 16.5	10.0 ± 13.4	11.8 ± 18.9	NS	NS	NS
Aortic PW	18.4 ± 15.1	18.4 ± 9.6	13.9 ± 12.1	NS	NS	NS
Aortic AW	17.6 ± 15.6	19.3 ± 11.7	14.8 ± 14.2	NS	NS	NS
Longitudinal velocity (cm/s)						
Aortic posterior sinus	−7.6 ± 2.0	−6.3 ± 2.9	−5.9 ± 2.5	NS	0.002	NS
Posterior ascending aorta	−5.9 ± 2.0	−6.1 ± 2.9	−5.4 ± 2.1	NS	NS	NS
Anterior ascending aorta	−5.6 ± 1.8	−6.4 ± 1.8	−4.8 ± 1.4	NS	NS	0.009
Anterior sinus	−5.0 ± 2.0	−5.8 ± 1.9	−4.3 ± 2.3	NS	NS	NS
Aortic PW	−6.8 ± 1.7	−6.2 ± 2.0	−5.6 ± 1.7	NS	0.01	NS
Aortic AW	−5.3 ± 1.6	−6.1 ± 1.7	−4.5 ± 1.5	NS	NS	0.01

*AW* anterior wall, *PW* posterior wall, *TD* transverse displacement, Values are mean (SD) unless otherwise indicated

The LD of an anterior aortic wall (both the sinus and ascending part) was greatest in group 1. Group 2 had significantly less LD of the anterior aortic wall than the controls or group 1. There was no statistically significant difference between the LD values of a posterior wall among the groups, but there was a tendency toward lower values in group 2 than in the controls or group 1.

Both the anterior and posterior walls of the ascending aorta during systole had statistically significantly greater TD in group 1 and the control group than in group 2. The largest TD of the posterior wall was recorded in group 1.

There was a tendency toward greater LD and TD values for the anterior aortic wall than the posterior wall in both DPAA groups. The largest LD values were observed in an anterior sinus segment and the smallest in the posterior aorta. In contrast, the least TD was seen in the aortic posterior sinus and the most in the anterior sinus.

There was a tendency toward lower LS in group 2, although the differences were not statistically significant. Interestingly, the LS in group 1was highest in the aortic posterior sinus and anterior ascending aorta segments compared with those of the controls but without statistical significance.

VL values for the anterior aortic wall were statistically significantly lower in group 2 than in group 1 and were lower than the control values without a significant difference. In contrast, the posterior aortic wall had lower VL values than the controls. VL values in the anterior sinus were greatest in group 1.

After excluding patients with severe aortic stenosis and severe aortic insufficiency from the analysis, we obtained similar results. There were no differences in the 2D-ST mechanical parameters between the controls and group 1. Less LD and TD of the anterior aortic wall and lower VL of the posterior sinus was found in group 2 than in the controls (*p* < 0.05). Lower LD values were found in the anterior aortic wall of the group 2 than in group 1 (*p* < 0.05).

We analyzed 2D-ST echocardiographic and stiffness parameters (aortic strain, elastic modulus, stiffness index β, distensibility) between patients with tricuspid or BAVs in both DPAA groups. No significant differences were found. Only LS of the aortic posterior sinus was higher in the BAV population (8.9 ± 10.4 vs. 18.8 ± 14.4, *p* = 0.02) (Table 4). The biomechanical aortic parameters (strain, distensibility) were reduced in group 2 compared with those in the controls and group 1. The elastic modulus and stiffness index β values were higher in group 2 than in the controls but without a statistically significant difference (Table 5).

**Table 4:** Comparison of echocardiographic and stiffness parameters between patients with tricuspid or bicuspid aortic valves in the two DPAA groups

Parameter	Tricuspid aortic valve	Bicuspid aortic valve	*p*
Aortic annulus (mm)	26.3 ± 2.8	28.4 ± 3.8	NS
Sinus at Valsalva level (mm)	48.9 ± 9.6	43.8 ± 6.5	NS
Sinotubular junction (mm)	43.8 ± 8.5	38.5 ± 4.9	0.02
Ascending aorta in end-diastole (mm)	50.8 ± 7.4	46.0 ± 5.0	0.02
Aortic annulus (mm/m^2^)	13.6 ± 1.4	14.2 ± 1.3	NS
Sinus at Valsalva level (mm/m^2^)	25.2 ± 5.0	22.0 ± 2.9	0.02
Sinotubular junction (mm/m^2^)	22.6 ± 4.3	19.3 ± 2.4	0.004
Ascending aorta in end-diastole (mm/m^2^)	26.6 ± 6.7	23.2 ± 3.1	NS
2D-ST echocardiographic parameters			
Longitudinal displacement (mm)			
Aortic posterior sinus	−4.6 ± 4.4	−4.7 ± 3.5	NS
Posterior ascending aorta	−3.5 ± 3.8	−2.5 ± 3.1	NS
Anterior ascending aorta	−6.7 ± 5.4	−9.5 ± 5.0	NS
Aortic anterior sinus	−10.0 ± 5.1	−11.7 ± 5.6	NS
Aortic PW	−4.0 ± 3.6	−3.9 ± 2.9	NS
Aortic AW	−8.3 ± 4.8	−10.5 ± 5.1	NS
Transverse displacement (mm)			
Aortic posterior sinus	3.1 ± 2.4	4.0 ± 2.6	NS
Posterior ascending aorta	4.0 ± 3.2	5.4 ± 1.6	NS
Anterior ascending aorta	−3.4 ± 2.9	−4.8 ± 2.9	NS
Aortic anterior sinus	−6.4 ± 4.2	−7.7 ± 3.5	NS
Aortic PW	4.2 ± 2.7	4.8 ± 2.0	NS
Aortic AW	−4.8 ± 2.5	−6.3 ± 3.1	NS
Longitudinal strain (%)			
Aortic posterior sinus	8.9 ± 10.4	18.8 ± 14.4	0.02
Posterior ascending aorta	13.7 ± 11.4	15.1 ± 11.5	NS
Anterior ascending aorta	21.2 ± 11.3	24.8 ± 16.3	NS
Aortic anterior sinus	11.5 ± 20.9	10.9 ± 12.7	NS
Aortic PW	11.4 ± 9.3	16.9 ± 11.3	NS
Aortic AW	15.1 ± 14.0	17.8 ± 12.1	NS
Longitudinal velocity (cm/s)			
Aortic posterior sinus	−5.9 ± 2.6	−5.8 ± 2.6	NS
Posterior ascending aorta	−5.6 ± 2.1	−6.1 ± 3.0	NS
Anterior ascending aorta	−5.2 ± 1.8	5.5 ± 1.3	NS
Anterior sinus	−4.7 ± 2.2	−4.8 ± 2.1	NS
Aortic PW	−5.8 ± 1.7	−6.0 ± 2.0	NS
Aortic AW	−4.9 ± 1.8	−5.2 ± 1.5	NS
Arterial stiffness parameters			
Aortic diameter change (mm)	2.5 ± 1.2	2.4 ± 1.4	NS
Aortic strain (%)	5.3 ± 2.8	5.6 ± 3.2	NS
Elastic modulus (mmHg)	19.8 ± 13.8	23.4 ± 29.6	NS
Stiffness index β	0.2 ± 0.1	0.2 ± 0.3	NS
Aortic distensibility (mmHg)^−1^	0.2 ± 0.1	0.2 ± 0.1	NS

*2D-ST* two-dimensional speckle tracking*, AW* anterior wall, *PW* posterior wall, Values are mean (SD) unless otherwise indicated

**Table 5:** Arterial stiffness parameters of the ascending aorta estimated by echocardiography

Parameter	Patient groups			*p*		
	Controls	Group 1 (≤45 mm)	Group 2 (>45 mm)	Controls vs. group 1	Controls vs. group 2	Group 1 vs. group 2
Aortic diameter change (mm)	1.9 ± 1.3	2.6 ± 1.3	2.2 ± 1.2	NS	NS	NS
Aortic strain (%)	6.3 ± 4.8	6.6 ± 3.4	4.4 ± 2.4	NS	0.028	0.03
Elastic modulus (mmHg)	13.1 ± 11.9	14.2 ± 10.6	23.9 ± 24.2	NS	NS	NS
Stiffness index β	0.1 ± 0.1	0.1 ± 0.1	0.2 ± 0.3	NS	NS	NS
Aortic distensibility (mmHg^−1^)	0.3 ± 0.3	0.2 ± 0.1	0.1 ± 0.1	NS	0.001	NS

Values are mean (SD) unless otherwise indicated

Correlations between the 2D-ST echocardiographic results and the arterial biomechanical parameters of the ascending aorta were calculated for the whole patient population. The LD of both the anterior and posterior aortic walls had a negative correlation with the aortic strain (*r* = −0.267 and *r* = −0.211). LD values were mathematically negative, and the lower LD was associated with less aortic strain.

Less aortic distensibility was associated with reduced LD and LS of the anterior aortic wall. The lower the LD of the anterior aortic wall, the worse was the estimated aortic distensibility.

The LS of the anterior aortic wall directly correlated positively with aortic strain and distensibility: the lower the LS, the lower were the aortic strain and distensibility (respectively, *r* = 0.22 and *r* = 0.283). No correlations between the VL and the stiffness parameters of the ascending aorta were observed (Table 6).

**Table 6:** Correlation between speckle-tracking echocardiographic and arterial stiffness parameters of the ascending aorta for the total study population

Parameter	Aortic strain	Aortic distensibility
Longitudinal displacement (mm)		
Aortic AW	−0.267**	−0.290**
Aortic PW	−0.211*	−0.185
Transverse displacement (mm)		
Aortic AW	−0.132	−0.169
Aortic PW	−0.059	−0.016
Longitudinal strain (%)		
Aortic AW	0.220*	0.283*
Aortic PW	0.076	0.105
Longitudinal velocity (cm/s)		
Aortic AW	−0.003	−0.047
Aortic PW	0.077	0.007

*AW* anterior wall, *PW* posterior wall

**Correlation is significant at the *p* < 0.01 level. *Correlation is significant at the *p* < 0.05 level

Because the 2D-ST-derived aortic wall motion parameters were higher in group 1 patients, the correlation between these parameters and the aortic diameters (including patients from both DPAA groups) were analyzed. The results are shown in Table 7. Negative correlations were found between the aortic dimensions and wall motion. That is, the higher/greater aortic dimensions were associated with reduced absolute values of the LD and TD of the posterior aortic wall and reduced LS of both the anterior and posterior aortic walls.

**Table 7:** Correlation between the speckle-tracking echocardiographic parameters and aortic diameters

Parameter	Aortic sinus diameter	Ascending aorta diameter	Aortic cross sectional area
Longitudinal displacement (mm)			
Aortic AW	0.23*	NS	NS
Aortic PW	0.22*	NS	NS
Transverse displacement (mm)			
Aortic AW	0.33**	0.28**	0.33**
Aortic PW	−0.22*	−0.37**	−0.41**
Longitudinal strain (%)			
Aortic AW	−0.23*	NS	NS
Aortic PW	−0.27*	NS	NS
Longitudinal velocity (cm/s)			
Aortic AW	NS	NS	NS
Aortic PW	NS	0.22*	NS

*AW* anterior wall, *PW* posterior wall

**Correlation is significant at the *p* < 0.01 level. *Correlation is significant at the *p* < 0.05 level

**Fig. 2:**
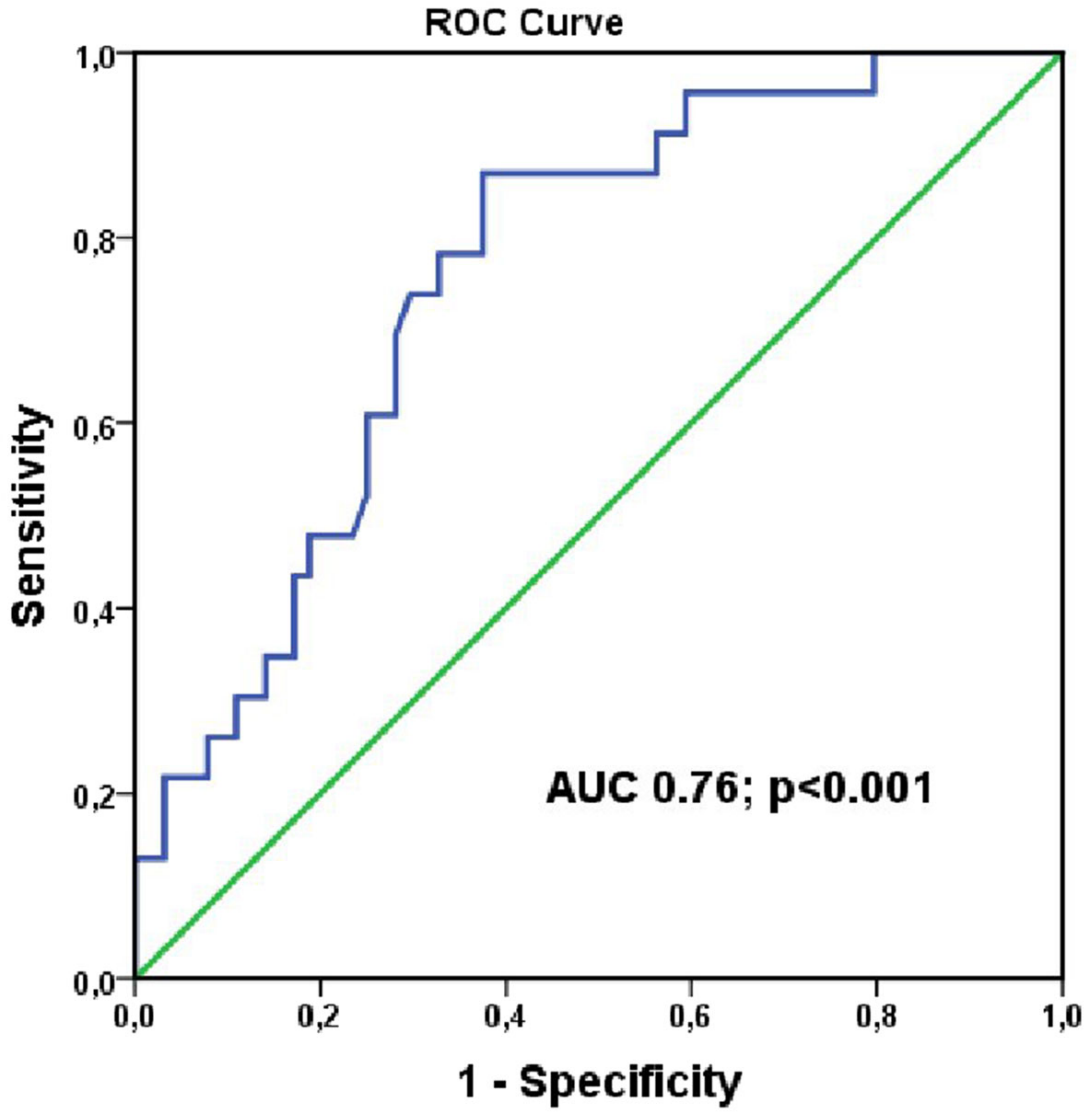
Receiver operating characteristic curve of transverse displacement of the aortic posterior wall, which predicts aortic dilation

ROC analysis showed that a value of 5.2 mm for TD of the posterior aortic wall [area under the curve (AUC) 0.76, *p* < 0.001, confidence interval (CI) 0.65–0.87; sensitivity 87%; specificity 63%] predicted aortic dilation >45 mm (Fig. 2).

## Discussion

For a long time, the most important determinant of ascending aorta rupture was the size of the aneurysm [13, 14]. More recent data, however, indicate that the absolute size may not be the only factor. Less aortic dilation may lead to dissection, whereas a larger aneurysm may remain stable. Thus, risk parameters for rupture, other than size, should be considered. Locally confined changes in aortic wall motion or an increase in local strain may be additional indicators [7].

Since 2008, 2D-ST studies have successfully assessed local aortic vascular wall properties [15]. We presented here a bedside approach to assessing human aortic wall biomechanics. We used the 2D strain imaging technique for evaluating LV myocardial strain and adapted it to imaging the ascending aorta. So far, we do not have sufficient data regarding the 2D-ST method for evaluating the ascending aorta to make definitive conclusions.

The main finding of our study was that the 2D-ST parameters of the ascending aorta were most impaired in patients with larger aortas (group 2, >45 mm aortic diameter). During systole, the ascending aorta stretches lengthwise, and all segments of the anterior aortic wall move forward from the aortic annulus. Longitudinal wall motion—i.e., LD of all of the anterior aortic wall and VL of both (anterior and posterior) walls—was diminished in group 2. There was a tendency toward lower LS values for both aortic walls in group 2. The TD of both aortic walls was also decreased in the larger-aorta group. Accordingly, LD of both aortic walls was greatest in group 1 (≤45 mm diameter).

Yurdakul et al. [16] demonstrated that the peak LS, the strain rate, and total LD values were significantly impaired in patients with BAVs, compared with the controls. In our study, only LS of the aortic posterior sinus was greater in the BAV population, with no significant difference between the other 2D-ST parameters. These results could have been influenced by the larger ascending aortic dimensions of tricuspid valve patients compared with those of the BAV patients. It seems that 2D-ST parameters are more dependent on the linear diameters of the aorta than on the type of aortic valve.

Strain imaging of the normal ascending and abdominal aortas, as well as their aneurysms, has demonstrated heterogeneous systolic strain distributions in all aortic segments [7]. Our study showed that four regions of the proximal aorta move differently. The largest LD values were observed in the anterior sinus segment, and the smallest were in the posterior ascending aorta. The TD was least in the aortic posterior sinus and most in the anterior sinus. We determined that a TD value of 5.2 mm is able to predict aortic dilation >45 mm.

The human aorta is often modeled mechanically as a hollow cylinder loaded with pulse pressure, resulting in a completely homogeneous strain distribution in the aortic wall [17]. During systole, the substantial proximal aortic axial displacement produces LS. Analyzing the LS parameters showed that there was a tendency toward lower LS rates in group 2, although we did not determine if there was a statistically significant difference.

We observed intermediate values among the 2D-ST data for the control group compared with those of groups 1 and 2 (i.e., values were lower than in group 1). We assumed that the ascending aorta at the beginning of the dilation process (to 45 mm) compensates for stretching during systole, and the biomechanical 2D-ST findings are thus higher than those for the controls.

In the present study, we measured aortic biomechanics by recording LS curves of the ascending aorta using 2D-ST imaging in addition to the 2D ultrasonographic images for arterial stiffness assessment. It is known that the elasticity of the aorta incorporate both - the property of dilating and the property of recoiling to its initial shape during systole and diastole. Also aortic stiffness describes the elastic resistance that the aorta sets against distension [18]. According to our data, significantly less aortic strain (aortic diameter change) and distensibility parameters of the ascending aorta were observed in the patients with aortas >45 mm. The elastic modulus and stiffness index β values were higher in group 2 than in the controls.

Oishi et al. adapted the same 2D-ST method for evaluating the abdominal aorta. Their study showed that the circumferential aortic strain and the strain rate were significantly lower, and the abdominal aortic stiffness parameter β was significantly greater, in older subjects than in the young/middle-aged group [15]. In our study, there were no age differences between the groups, and the stiffness index β and elastic modulus were greatest in group 2.

In the Yurdacul et al. study, aortic strain and distensibility were significantly impaired in patients with BAVs, compared with that of the control group, and aortic stiffness was markedly increased in the patients with BAVs [16]. We did not observe statistically significant differences between the tricuspid and BAV patients in regard to stiffness parameters of the ascending aorta. This lack of a great difference could be due to smaller aortic dimensions in the BAV group and a higher rate of aortic insufficiency in this group. We established higher LS values for the aortic posterior sinus only in BAV patients.

The same findings were presented in the Ozdemir study of hypertensive non-dipper patients, but the diameters of the ascending aorta were not described [19]. In our study, more of the group 2 patients had a history of systemic hypertension, although the diastolic blood pressure was lower than that in the controls. Approximately half of the patients in both DPAA groups had aortic regurgitation. This group showed increased stiffness and reduced distensibility and strain of the ascending aorta. These changes could result from aortic dilation and arterial hypertension. Both factors seem to be responsible for changes in aortic elastic properties.

Recently, more attention has been paid to complex imaging modalities, such as computed tomography (CT) and magnetic resonance imaging (MRI), especially in regard to imaging the aortic arch and descending aorta, because of the methodological limitations of echocardiography. It is important to note that the echocardiographic data concerning aortic biomechanics correlated with the CT and MRI results. Although CT and MRI have the advantage of being noninvasive, they remain expensive, and the availability of scanning facilities is limited.

We obtained weak to moderate correlation between the 2D-ST indices and the aortic stiffness parameters and aortic dimensions. A wider aorta was stiffer and was associated with lower absolute values for wall motion in longitudinal and transverse directions. This finding confirms that the dilated aorta cannot produce normal movement during several cardiac cycles, which could be a predictor of possible mechanical complications.

## Study limitations

Our study had some limitations. About 20% of all of the investigated persons were not included in the study because of a poor acoustic window. In the cases of inadequate quality of echocardiographic images, the software could not perform SP analysis properly, which reduced the ability to perform analysis in all the patients. The off-line software for the ST images was created for analyzing the left ventricle, so this effort is simply a pilot study of 2D-ST imaging of the aorta.

We analyzed only the feasibility of 2D-ST echocardiography as a tool for assessing aortic biomechanics. We did not expand our work to determine if it could provide a prognosis for patients with aortic dilation or dissection.

In the present study we investigated a small group of patients, but we presume that a larger size of sample would show more precise results.

## Conclusions

Both DPAA groups (ascending aortic diameters >40 mm) had reduced elasticity and increased stiffness of the ascending aorta. The most marked changes of the biomechanical parameters were seen in patients whose ascending aortic diameter was >45 mm. The longitudinal wall motion of the ascending aorta was mostly impaired in patients whose aorta was >45 mm (i.e., LD of the anterior aortic wall and LV of the posterior and anterior walls). TD of the posterior and anterior aortic walls also occurred statistically significantly less in patients whose aortic diameter was >45 mm. A TD value of 5.2 mm predicts aortic dilation >45 mm (AUC 0.76, *p* < 0.001, CI 0.65–0.87; sensitivity 87%; specificity 63%).

More extensive aortic dilation is associated with reduced aortic parameters (strain, distensibility) and increased elastic modulus and stiffness index β values. Lower LD and LS values were associated with less aortic strain and distensibility.

2D-ST echocardiography can be applied in daily clinical practice for evaluating aortic biomechanics.

## Abbreviations

2D-ST: Two-dimensional speckle-tracking echocardiography
BAV: Bicuspid aortic valve
DBP: Diastolic blood pressure
DD: Diastolic aortic diameter
DPAA: Dilative pathology of the ascending aorta
EF: Ejection fraction
LD: Longitudinal displacement
LS: Longitudinal strain
LV: Left ventricle
SBP: Systolic blood pressure
SD: Systolic aortic diameter
STJ: Sinotubular junction
TD: Transversal displacement
VL: Longitudinal velocity
